# Causal effects of lipid-lowering therapies on aging-related outcomes and risk of cancers: a drug-target Mendelian randomization study

**DOI:** 10.18632/aging.205347

**Published:** 2023-12-19

**Authors:** Han Chen, Xinyu Tang, Wei Su, Shuo Li, Ruoyun Yang, Hong Cheng, Guoxin Zhang, Xiaoying Zhou

**Affiliations:** 1 Department of Gastroenterology, The First Affiliated Hospital of Nanjing Medical University, Nanjing, Jiangsu, China; 2 Department of Radiation Oncology, The First Affiliated Hospital of Nanjing Medical University, Nanjing, Jiangsu, China; 3 Department of Neurology, The First Affiliated Hospital of Nanjing Medical University, Nanjing, Jiangsu, China

**Keywords:** lipid-lowering drugs, aging, Mendelian randomization

## Abstract

Background: Despite the widespread use of statins, newer lipid-lowering drugs have been emerging. It remains unclear how the long-term use of novel lipid-lowering drugs affects the occurrence of cancers and age-related diseases.

Methods: A drug-target Mendelian randomization study was performed. Genetic variants of nine lipid-lowering drug-target genes (*HMGCR, PCKS9, NPC1L1, LDLR, APOB, CETP, LPL, APOC3*, and *ANGPTL3*) were extracted as exposures from the summary data of Global Lipids Genetics Consortium Genome-Wide Association Studies (GWAS). GWAS summary data of cancers and noncancerous diseases were used as outcomes. The inverse-variance weighted method was applied as the main statistical approach. Sensitivity tests were conducted to evaluate the robustness, pleiotropy, and heterogeneity of the results.

Results: In addition to marked effects on decreased risks of atherosclerotic cardiovascular diseases, genetically proxied lipid-lowering variants of *PCKS9*, *CETP*, *LPL*, *LDLR*, and *APOC3* were associated with longer human lifespans (*q<0.05*). Lipid-lowering variants of *ANGPTL3* and *LDLR* were associated with reduced risks of colorectal cancer, and *ANGPTL3* was also associated with lower risks of gastric cancer (*q<0.05*). Lipid-lowering *LPL* variants were associated with decreased risks of hypertension, type 2 diabetes, nonalcoholic fatty liver disease, and bladder cancer (*q<0.05*). Lipid-lowering variants of *PCKS9* and *HMGCR* were associated with decreased risks of osteoporosis (*q<0.05*). Lipid-lowering *APOB* variants were associated with a decreased risk of thyroid cancer (*q<0.05*).

Conclusions: Our study provides genetic evidence that newer nonstatin lipid-lowering agents have causal effects on decreased risks of several common cancers and cardiometabolic diseases. These data provide genetic insights into the potential benefits of newer nonstatin therapies.

## INTRODUCTION

Statins are commonly used lipid-lowering drugs that target 3-hydroxy-3-methylglutaryl coenzyme A reductase (*HMGCR*) [1]. As first-line agents to lower plasma LDL cholesterol (LDL-C), statins have shown consistent benefits in both primary and secondary prevention for atherosclerotic cardiovascular disease (ASCVD) as supported by numerous clinical trials [2, 3]. However, even with optimal statin therapy, there are still notable residual ASCVD risks [4]. In addition, statin adherence is not clinically sufficient due to a potential risk of newly diagnosed type 2 diabetes [5] and intolerance of adverse events such as myopathy and hepatopathy [6]. Therefore, to improve lipid-lowering effects, new nonstatin lipid-lowering pharmaceutical agents have emerged.

In recent years, several gene-target drugs have been developed due to their favorable lipid-lowering effect. Cholesterol absorption inhibitors, proprotein convertase subtilisin/kexin type 9 (*PCSK9*) inhibitors, and bempedoic acid (*BA*) are several newer nonstatin medications with cholesterol-lowering effects [7, 8], while angiopoietin-like protein 3 (*ANGPTL3*) inhibitors and antisense oligonucleotides targeting the mRNA of apoprotein C-III (*APOC3*) have effects on lowering serum triglyceride levels [9]. As clinical trials investigating the effectiveness and safety of several newer nonstatin drugs are still currently in progress [10], the impact of long-term use of these drugs on morbidity and mortality remains unclear. Therefore, in our study, we aim to investigate the genetic impact of different lipid-lowering drugs on cardiometabolic diseases, the risk of cancers, and age-related outcomes by examining gene targets.

Mendelian randomization (MR) is an analytical approach that has been widely applied to investigate the causal relations between exposures and outcomes [11]. A drug-target MR analysis belongs to the MR study but only retains genetic variants in or near the target gene of the drug substance [12]. The effects of genetic variants within the encoding gene of a drug target can illustrate the potential causal effect of controlling a drug target on modulating exposures and outcomes. Therefore, we performed a two-sample MR study with drug-target MR analysis, mimicking the long-term administration of different lipid-lowering agents in randomized clinical trials, to provide genetic insights into the safety of different lipid-lowering drugs on age-related traits.

## MATERIALS AND METHODS

### Study design and data sources

Our MR study followed the Strengthening the Reporting of Observational Studies in Epidemiology using Mendelian Randomization (STROBE-MR) guidelines (Supplementary Table 1). A two-sample MR analysis with drug-target analysis was designed. The exposures comprised LDL-cholesterol- or triglyceride-lowering genetic variants in or near various drug-target genes. The classification of lipid-lowering drugs and their target genes was based on the latest expert consensus and guidelines regarding lipid-lowering therapies [10, 13], which are summarized in Table 1. To enhance the credibility of the causal effects of gene variants, we performed positive control analyses, given the recognized benefits of lipid-lowering drugs in coronary artery disease.

**Table 1:** Summary of genetically proxied lipid-lowering drug targets.

**Drug effect**	**Drug class**	**Drug target**	**Encoding genes**	**Gene location** **(GRCh37 from ensembl)**	**Drug substance**	**Eligible IVs**
↓LDL-C	Key Modulator	LDL Receptor	*LDLR*	CHR:19:11,200,038-11,244,492	-	yes
	HMGCR inhibitors	HMG-CoA reductase	*HMGCR*	CHR:5:74,632,154-74,657,929	Atorvastatin Rosuvastatin etc.	yes
	ACLY inhibitors	ATP-citrate synthase	*ACLY*	CHR:17:40,023,161-40,086,795	Bempedoic acid	no
	PCSK9 inhibitors	Proprotein Convertase Subtilisin/Kexin Type 9	*PCSK9*	CHR:1:55,505,221-55,530,525	Evolocumab Alirocumab	yes
	TC absorption inhibitors	Niemann-Pick C1-like 1	*NPC1L1*	CHR:7:44,552,134-44,580,914	Ezetimibe	yes
	ASO targeting ApoB mRNA	Apo B100	*APOB*	CHR:2:21,224,301-21,266,945	Mipomersen	yes
	ASO targeting CETP mRNA	Cholesteryl Ester Transfer Protein	*CETP*	CHR:16:56,995,762-57,017,757	Torcetrapib	yes
	BA sequestrants	Bile acids	*-*	-	Cholestyramine Colestipol	no
↓TG	Key Modulator	Lipoprotein Lipase	*LPL*	CHR:8:19,759,228-19,824,769	-	yes
	Fibrates	Peroxisome Proliferator-Activated Receptor-alpha	*PPARA*	CHR:22:46,546,424-46,639,653	Fenofibrate Gemfibrozil	no
	ANGPTL3 inhibitors	Angiopoietin-related protein 3	*ANGPTL3*	CHR:1:63,063,158-63,071,830	Evinacumab	yes
	ASO targeting ApoC-III mRNA	Apo C-III	*APOC3*	CHR:11:116,700,422-116,703,788	Volanesorsen	yes

Abbreviations: LDL-C, Low-Density Lipoprotein Cholesterol; TG, Total triglyceride; LDLR, Low-Density Lipoprotein Receptor; HMGCR, 3-hydroxy-3-methylglutaryl coenzyme A reductase; PCKS9, Proprotein Convertase Subtilisin/Kexin Type 9; NPC1L1, Niemann-Pick C1-like 1; APOB, Apoprotein B-100; CETP, Cholesteryl Ester Transfer Protein; LPL, Lipoprotein Lipase; ANGPTL3, Angiopoietin-related protein 3; APOC3, Apoprotein C-III.

The outcome data included multiple GWAS summary data of cardiometabolic diseases (coronary atherosclerosis, major coronary heart disease events, hypertension, type 2 diabetes, nonalcoholic fatty liver disease (NAFLD)), risk of cancers (colorectal cancer, gastric cancer, esophageal cancer, hepatocellular carcinoma, pancreatic cancer, lung cancer, thyroid cancer, bladder cancer, and cerebral tumors), and age-related outcomes (parental lifespan and longevity, telomere length, chronic obstructive pulmonary disease (COPD), Alzheimer's disease/dementia, and osteoporosis). All the data sources used in this study were derived from publicly accessible GWAS summary data of European populations, and detailed information is presented in Table 2.

**Table 2:** Data resources of the exposures and outcomes used in this study.

**GWAS traits**	**GWAS consortium**	**First author**	**Year**	**PMID**	**Population**	**Data type**	**Sample size**	**Case/control**	**Unit**
**Circulating Lipids**									
LDL cholesterol	GLGC	Willer CJ	2013	24097068	96% European	Continuous	173,082	-	SD (mg/dL)
Total cholesterol	GLGC	Willer CJ	2013	24097068	96% European	Continuous	187,365	-	SD (mg/dL)
Total Triglycerides	GLGC	Willer CJ	2013	24097068	96% European	Continuous	177,861	-	SD (mg/dL)
**Age-related Outcomes**									
Parental lifespan	UKBiobank	Timmers PR	2019	30642433	European	Continuous	500,193	-	SD
Telomere length	UKBiobank	Codd V	2021	34611362	European	Continuous	472,174	-	SD
Chronic obstructive pulmonary disease	MRC-IEU	Ben Elsworth	2018	-	European	Binary	462,933	1,605/461,328	LogOR
Alzheimer's disease/dementia	MRC-IEU	Ben Elsworth	2018	-	European	Binary	399,793	19,255/380,538	LogOR
Osteoporosis	MRC-IEU	Ben Elsworth	2018	-	European	Binary	462,933	7,547/455,386	LogOR
**Cardiometabolic Diseases**									
Coronary atherosclerosis	FinnGen	-	2022	-	European	Binary	328,042	42,421 / 285,621	LogOR
Major coronary heart disease events	FinnGen	-	2022	-	European	Binary			LogOR
Hypertension	MRC-IEU	Ben Elsworth	2018	-	European	Binary	463010	54,358/408,652	LogOR
Type 2 Diabetes	DIAMANTE	Mahajan A	2018	30297969	European	Binary	898,130	74,124 / 824,006	LogOR
Nonalcoholic fatty liver disease		Namjou B	2019	31311600	European	Binary	9677	1,106/8,571	LogOR
**Maligant Tumors**									
Colorectal cancer	GECCO	Fernandez-Rozadilla C	2023	36539618	European	Binary	185,616	78,473/107,143	LogOR
Gastric cancer	FinnGen	-	2022	-	European	Binary	260810	1,227/259,583	LogOR
Esophageal cancer	FinnGen	-	2022	-	European	Binary	260086	503/259,583	LogOR
Pancreatic cancer	FinnGen	-	2022	-	European	Binary	260832	1,249/259,583	LogOR
Hepatocelullar cancer	FinnGen	-	2022	-	European	Binary			LogOR
Lung cancer	UKBiobank	Burrows K	2021	-	European	Binary	374687	2,761/372,016	LogOR
Thyroid cancer	FinnGen	-	2022	-	European	Binary	261108	1,525/259,583	LogOR
Bladder cancer	FinnGen	-	2022	-	European	Binary	259667	84/259,583	LogOR
Brain tumors	FinnGen	-	2022	-	European	Binary	260357	774/259,583	LogOR

**Figure 1:**
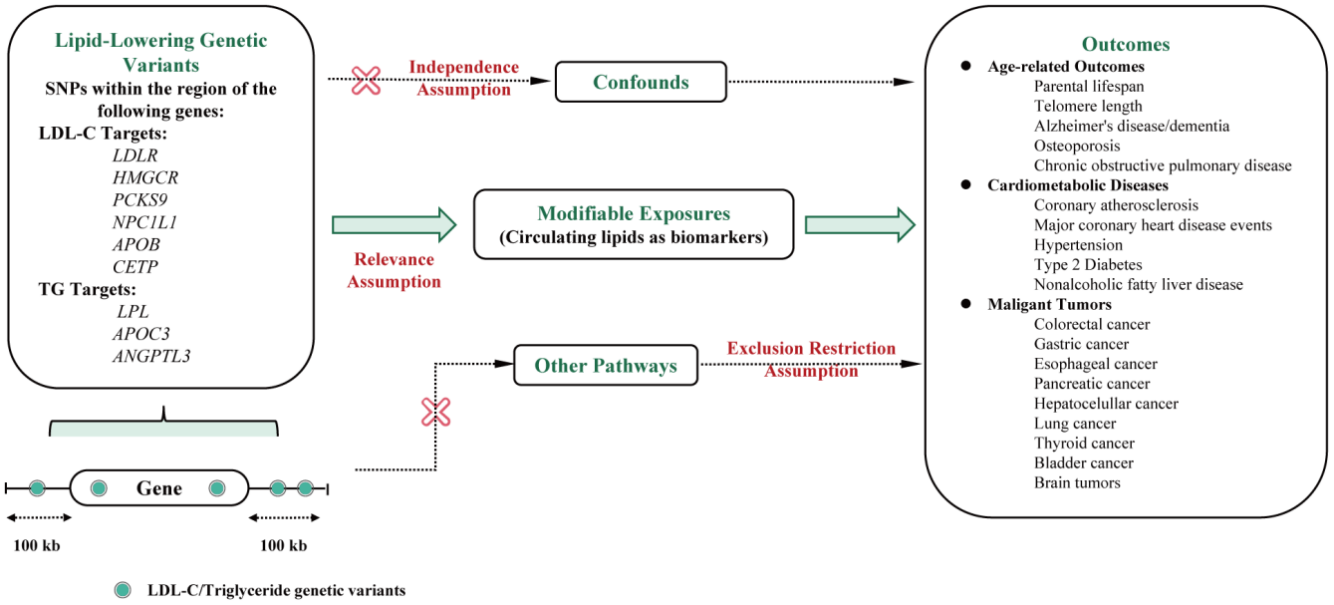
**Flowchart of the study design and MR assumptions.** Assumptions of the Mendelian randomization study: in this study, genetic instruments were selected to represent the pharmacological modulation of drug target proteins based on their associations with circulating lipid concentrations (relevance assumption). Additionally, it was assumed that the selected genetic variants are not associated with confounding factors (independence assumption). The third assumption was that genetic variants should not affect human lifespan through other pathways (exclusion restriction assumption). Abbreviations: LDL-C, Low-Density Lipoprotein Cholesterol; TC, Total cholesterol; TG, Total triglyceride; LDLR, Low-Density Lipoprotein Receptor; HMGCR, 3-hydroxy-3-methylglutaryl coenzyme A reductase; PCKS9, Proprotein Convertase Subtilisin/Kexin Type 9; NPC1L1, Niemann-Pick C1-like 1; APOB, Apoprotein B-100; CETP, Cholesteryl Ester Transfer Protein; LPL, Lipoprotein Lipase; ANGPTL3, Angiopoietin-related protein 3; APOC3, Apoprotein C-III; CHD, Major coronary heart disease; CAS, Coronary atherosclerosis; T2D, Type 2 diabetes.

Figure 1 presents the main MR assumptions. As the main effect of lipid-lowering drugs is to reduce LDL-C or triglyceride levels, we used the associations of these selected genetic instruments with circulating lipid concentrations to proxy the pharmacological modulation of the drug-target protein (relevance assumption). The assumption is that genetic variants are not associated with confounders (independence assumption) and affect human lifespan through other pathways (exclusion restriction assumption) [14]. This study employed publicly available summary statistics for analysis, and no ethical approval was needed.

### Selection of genetic variants

For drug-target MR, information on pharmacologically active protein targets and their encoding genes was extracted from the DrugBank (https://go.drugbank.com/) and NCBI Gene Database (https://www.ncbi.nlm.nih.gov/gene/). A total of 11 target genes were identified, including low-Density Lipoprotein Receptor (*LDLR)*, 3-hydroxy-3-methylglutaryl coenzyme A reductase (*HMGCR)*, ATP-citrate synthase (*ACLY*), proprotein Convertase Subtilisin/Kexin Type 9 (*PCKS9*), Niemann-Pick C1-like 1 (*NPC1L1*), apoprotein B-100 (*APOB*), cholesteryl ester transfer protein (*CETP*), *lipoprotein lipase* (*LPL)*, peroxisome proliferator activated receptor alpha (*PPARA*), angiopoietin-related protein 3 (*ANGPTL3*), and apoprotein C-III (*APOC3*). Genetic variants within these genes that encode protein targets of lipid-lowering drugs (cis-variants) were extracted from the GWAS summary data from the Global Lipids Genetics Consortium [15]. Cis-variants are defined as genetic variants located on the same DNA molecule as the target gene [16]. The serum levels of LDL-C and TG were used as proxies for LDL-C-lowering and TG-lowering targets, respectively. Drug-target SNPs, clumped to an LD threshold of *r^2^<0.3* with a 100 kb window distance, were identified at a genome-wide level of significance (*p≤5×10^−8^*) within ±100 kb regions of the corresponding genes. *PPARA* and *ACLY* were excluded from further analysis due to insufficient numbers of SNPs identified as drug proxies. Steiger filtering [17] was also used to identify the bidirectional effects, and variants with reverse causal effects were removed accordingly. SNPs with inconsistent alleles (i.e., A/G vs. A/C) were strictly excluded.

### Statistical analysis

The inverse variance weighting (IVW) method [18] was primarily used to estimate the causal effects. This approach estimates the causality of a 1 standard deviation increase in exposure to genetic predictors of outcome. Beta estimates were utilized to evaluate GWAS data with continuous outcomes, while odds ratios were used to estimate the GAWS data with binary outcomes. The association estimates of the same trait were combined using a meta-analysis of the fixed or random effects model based on heterogeneity [19].

To test the MR assumptions in the study design, we first calculated the F statistic for each instrument using the following formula: F=R^2^(n−1−k)/(1−R^2^)k, (R^2^ stands for the proportion of variation explained, k stands for the number of eligible SNPs, and n stands for the sample size) [20]. No significant weak instrumental bias was defined as eligible SNPs with F-statistics greater than 10. Statistical power was estimated using the mRnd website (https://shiny.cnsgenomics.com/mRnd/). To validate the results from the IVW method, we conducted sensitivity tests using MR-Egger regression, weighted median, maximum likelihood, and weighted mode methods [21]. We estimated heterogeneity and horizontal pleiotropy using the Cochran Q test and MR Egger’s intercept test. Additionally, we utilized “leave-one-out” analysis to identify heterogeneous SNPs by omitting each instrumental SNP in turn. *P*-values were adjusted from multiple testing using the false discovery rate (FDR*, q-value*) with the Benjamin-Hochberg method.

All analyses were implemented in R software version 4.1.0 using the TwosampleMR (github.com/MRCIEU/TwpSampleMR), MendelianRandomiszation, and coloc R packages. Forest plots were derived from the ggplot and ggplot2 R packages, and heatmaps were derived from the pheatmap R packages. The figure illustrating the pharmacological mechanisms of lipid-lowering drugs was drawn using the FigDraw platform (https://www.figdraw.com/).

### Availability of data and materials

All data generated or analysed during this study are included in this published article and its Supplementary Information Files. The availability of all the data used in the study was summarized in Supplementary Table 2.

### Consent for publication

This manuscript has not been previously published. All authors have consented to the publication of the manuscript in this journal.

## RESULTS

### Effects of genetic variation in lipid-lowering drug targets on human lifespan

**Figure 2:**
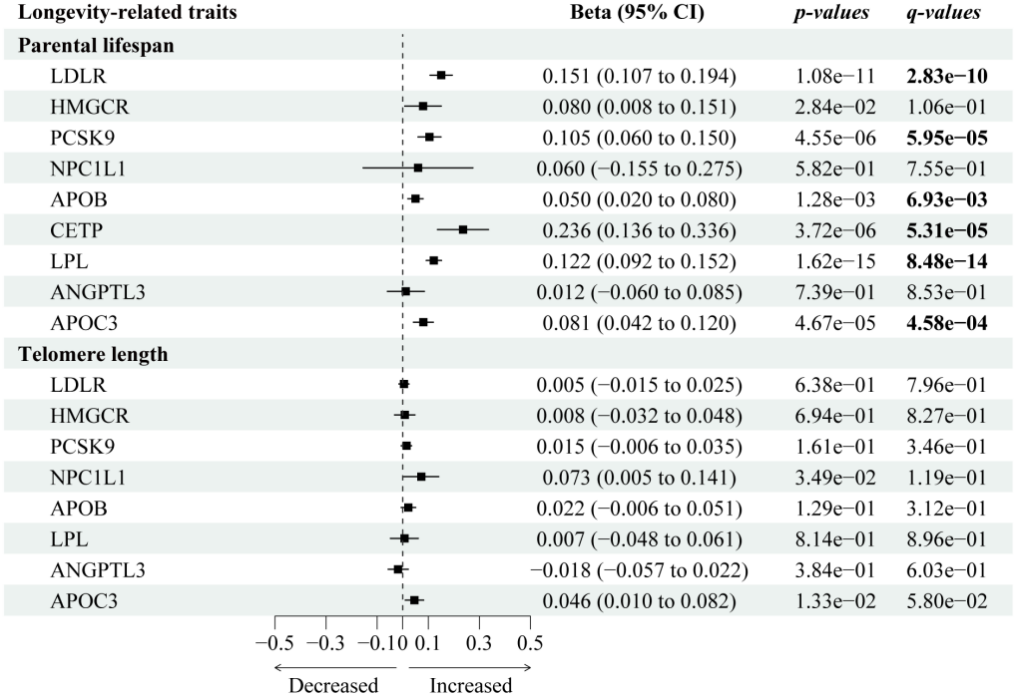
**Forest plot visualizing the causal effects of the genetically proxied lipid-lowering drug targets on longevity-related traits.** The forest plot showed the estimated effects of 1 mmol/L lower LDL-C or TG concentration by target-specific variants in each drug target gene on longevity-related traits, using the IVW method. Beta and 95% CI were used in quantitative outcomes.

We used the largest GWAS dataset that contains the largest-scale lifespan-associated GWAS summary data among 500,193 European individuals [22]. Figure 2 presents the causal effects of 9 genetically proxied lipid-lowering gene targets on human lifespan or longevity-related traits. After Steiger filtration, 13 variants were selected to proxy *LDL* lowering through *LDLR* modulator, 7 for *HMGCR*, 12 for *PCKS9*, 3 for *NPC1L1*, 21 for *LPL*, 18 for *APOB*, 4 for *CETP*, 4 for *ANGPTL3*, and 10 for *APOC3*, with all F statistics greater than 10 (Supplementary Table 2).

We identified six lipid-lowering variants that were associated with increased lifespan, including *PCKS9* (Beta 0.11; 95% CI: 0.06 to 0.15; *p-_IVW_*=4.55×10^-6^, *FDR*=5.95×10^-5^), *CETP* (Beta 0.24; 95% CI: 0.14 to 0.34; *p-_IVW_*=3.72×10-06, *FDR*=5.31×10^-5^), *APOC3* (Beta 0.08; 95% CI: 0.04 to 0.12; *p-_IVW_*=4.67×10^-5^, *FDR*=4.58×10^-4^), *LDLR* (Beta 0.15; 95% CI: 0.11 to 0.19; *p-_IVW_*=1.08×10^−11^, *FDR*=2.83×10^-10^), and *LPL* (Beta 0.12; 95% CI: 0.09 to 0.195; *p-_IVW_*=1.62×10^-15^, *FDR*=8.48×10^-14^). There was little statistical evidence of longevity-associated effects among *NPC1L1* (Beta 0.06; 95% CI: -0.16 to 0.28; *p-_IVW_*=0.58, *FDR*=0.76) and *HMGCR* (Beta 0.08; 95% CI: 0.01 to 0.15; *p-_IVW_*=0.03, *FDR*=0.11). Sensitivity tests (Supplementary Table 3) showed consistent trends in the estimates, with no statistical evidence of bias from horizontal pleiotropy and heterogeneity (Supplementary Table 4).

### Results of positive control analysis

**Figure 3:**
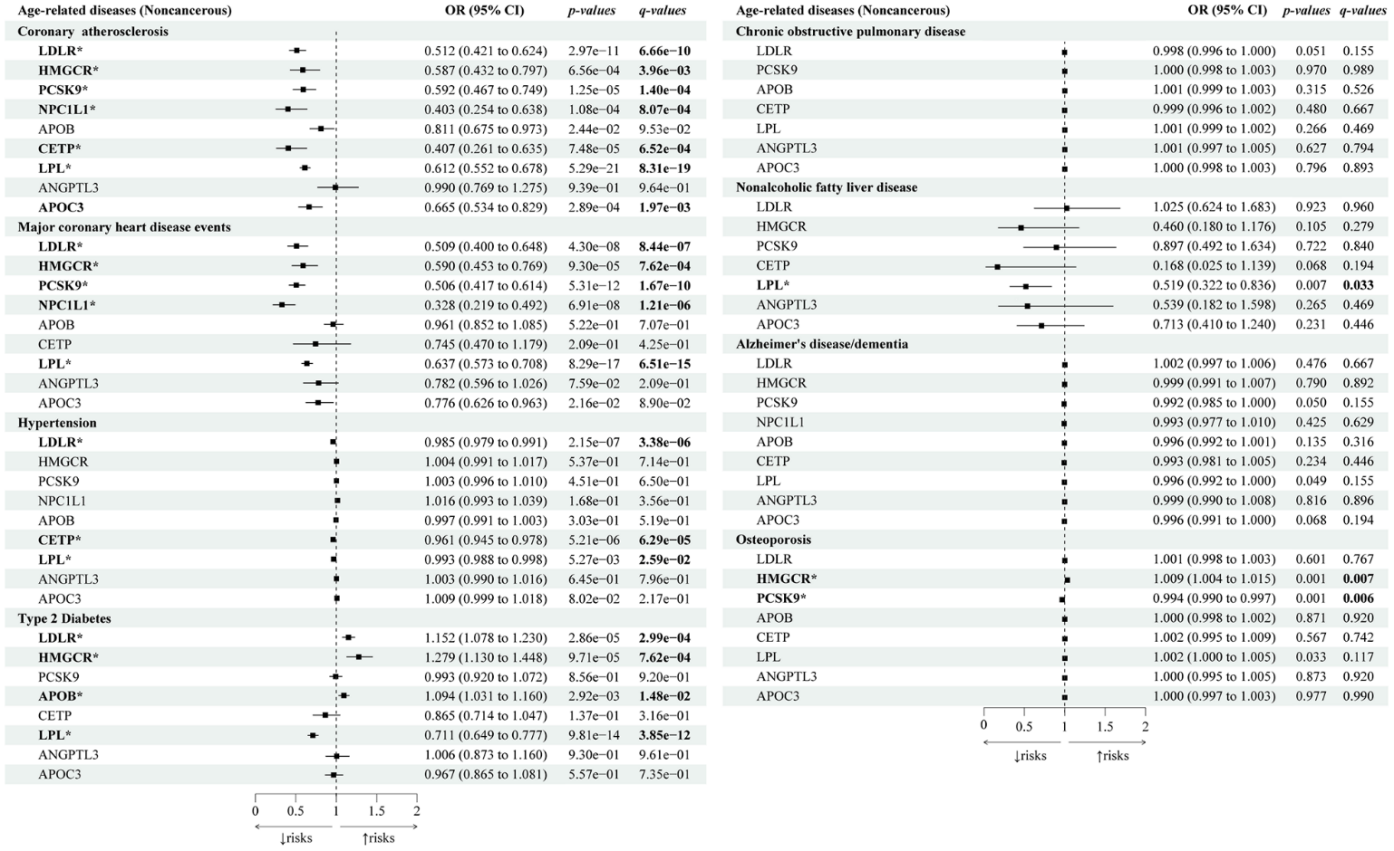
**Causal effects of the genetically proxied lipid-lowering drug targets on age-related noncancerous diseases.** The forest plot showed the estimated effects of 1 mmol/L lower LDL-C or TG concentration by target-specific variants in each drug target gene on age-related noncancerous diseases, using the IVW method. OR and 95% CI indicated the effect estimates of a 1mmol/L change of circulating lipids on outcomes.

In the positive control analysis (Figure 3), we identified significant associations between most lipid-lowering gene targets (*HMGCR, LDLR, PCSK9, NPC1L1, and LPL*) and a decreased risk of both coronary atherosclerosis and major coronary heart disease (CHD) events, except for *APOB*, *CETP,* and *APOC3* (only associated with reduced coronary atherosclerosis risks) and *ANGPTL3* (no association with either CHD or coronary atherosclerosis).

### Lipid-lowering drug targets and age-related noncancerous diseases

We also investigated the genetic association between different lipid-lowering drugs and cardiometabolic diseases. In addition to marked effects on decreased risks of atherosclerotic cardiovascular diseases in positive control analysis, lipid-lowering variants of *CETP* (OR 0.41; 95% CI: 0.26 to 0.64; *p-IVW*=7.48×10^-5^, *FDR*=6.52×10^-4^) and *LPL* (OR 0.61; 95% CI: 0.55 to 0.68; *p-IVW*=5.27×10^-3^, *FDR*=0.03) were associated with decreased risks of hypertension. Genetically proxied lipid-lowering variants of *LDLR* (OR 1.15; 95% CI: 1.08 to 1.23; *p-IVW*=2.86×10^-5^, *FDR*=2.99×10^-4^)*, HMGCR* (OR 1.29; 95% CI: 1.13 to 1.45; *p-IVW*=9.71×10^-5^, *FDR*=7.62×10^-4^), and *APOB* (OR 1.09; 95% CI: 1.16 to 1.03; *p-IVW*=2.92×10^-3^, *FDR*=1.48×10^-2^) were associated with increased risks of T2D. Lipid-lowering variants of *LPL* were associated with decreased risks of both T2D (OR 0.71; 95% CI: 0.65 to 0.77; *p-IVW*=9.81×10^-3^, *FDR*=3.85×10^-12^) and NAFLD (OR 0.52; 95% CI: 0.32 to 0.84; *p-IVW*=7.02×10^-3^, *FDR*=3.34×10^-2^). Lipid-lowering variants of *HMGCR* (OR 1.01; 95% CI: 1.004 to 1.02; *p-IVW*=1.39×10^-3^, *FDR*=7.27×10^-3^) were associated with a slightly increased risk of osteoporosis whereas *PCKS9* (OR 0.99; 95% CI: 0.99 to 0.997; *p-IVW*=9.89×10^-4^, *FDR*=5.75×10^-3^) was associated with decreased risks of osteoporosis. In addition, we found that none of the nine drug-target genes were associated with COPD and Alzheimer's disease/dementia (with all q-values>0.05). Sensitivity tests showed consistent trends in the estimates, with no statistical evidence of bias from horizontal pleiotropy and heterogeneity (Supplementary Tables 5, 6).

### Lipid-lowering drug targets and risk of cancers

**Figure 4:**
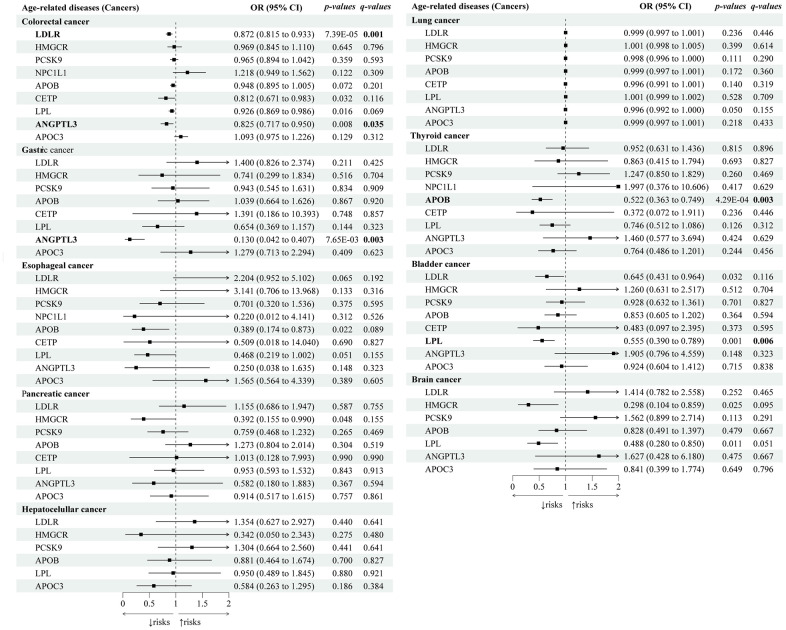
**Causal effects of the genetically proxied lipid-lowering drug targets on cancers.** The forest plot showed the estimated effects of 1 mmol/L lower LDL-C or TG concentration by target-specific variants in each drug target gene on cancers, using the IVW method. OR and 95% CI indicated the effect estimates of a 1mmol/L change of circulating lipids on outcomes.

Next, we explored the genetic associations between lipid-lowering drugs and cancers (Figure 4). We identified that lipid-lowering variants of *ANGPTL3* (OR 0.83; 95% CI: 0.72 to 0.95; *p-IVW*=7.65×10^-3^, *FDR*=3.53×10^-2^) and *LDLR* (OR 0.83; 95% CI: 0.82 to 0.93; *p-IVW*=7.39×10^-5^, *FDR*=6.52×10^-4^) were associated with decreased risks of colorectal cancers, and *ANGPTL3* (OR 0.13; 95% CI: 0.04 to 0.41; *p-IVW*=4.64×10^-4^, *FDR*=2.91×10^-3^) was also associated with lower risks of gastric cancers. Lipid-lowering *LPL* variants were associated with decreased risks of bladder cancers (OR 0.56; 95% CI: 0.39 to 0.79; *p-IVW*=1.06×10^-3^, *FDR*=5.94×10^-3^). Lipid-lowering *APOB* variants were associated with a decreased risk of thyroid cancer (OR 0.52; 95% CI: 0.36 to 0.75; *p-IVW*=4.29×10^-4^, *FDR*=2.81×10^-3^). None of the nine drug-target genes were genetically associated with esophageal cancers, pancreatic cancers, hepatocellular cancers, lung cancers, or brain cancers (with all q-values>0.05). Sensitivity tests showed consistent trends in the estimates, with no statistical evidence of bias from horizontal pleiotropy and heterogeneity (Supplementary Tables 7, 8).

**Figure 5:**
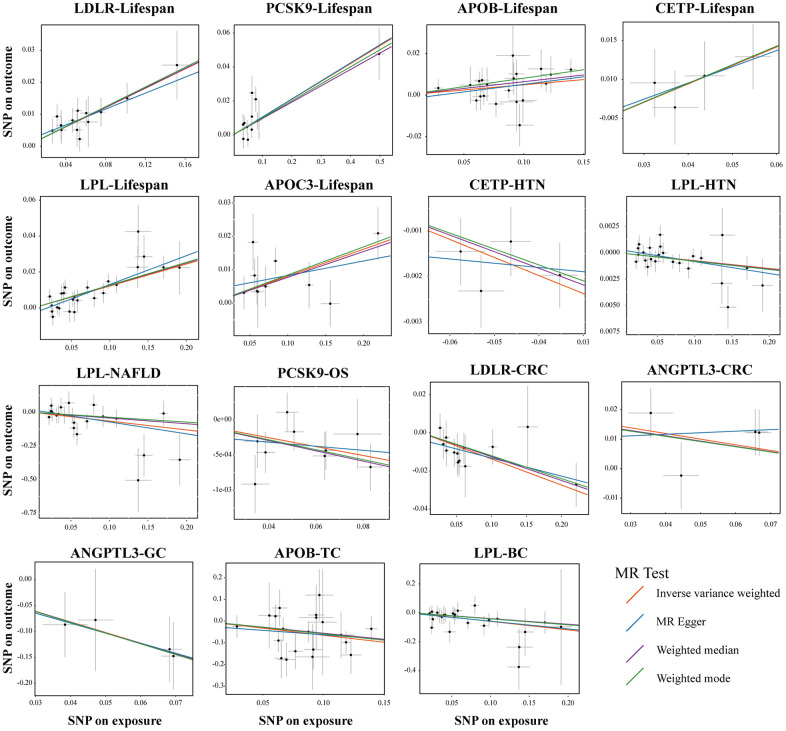
**Sensitivity test in drug-target MR analyses.** Scatter plots of four statistical tests showing representative lipid-lowering drug target genes that had a causal relationship on the different outcomes. Each black dot represents an SNP significantly associated with lipid-lowering effects. The gray lines around the dot represent the 95% confidence intervals of each SNP. Four lines generated by different MR tests were colored as red (Inverse Variance Weighted, IVW), blue (MR Egger), purple (Weighted Median), and green (Weighted mode). the X-axis represents the SNPs effects of certain lipid-lowering genes, and the Y-axis represents the SNPs effects of different outcomes. Abbreviations: HTN, hypertension; NAFLD, non-alcoholic fatty liver disease; OS, osteoporosis; CRC, colorectal cancer; GC, gastric cancer; TC, thyroid cancer, BC, bladder cancer.

**Figure 6:**
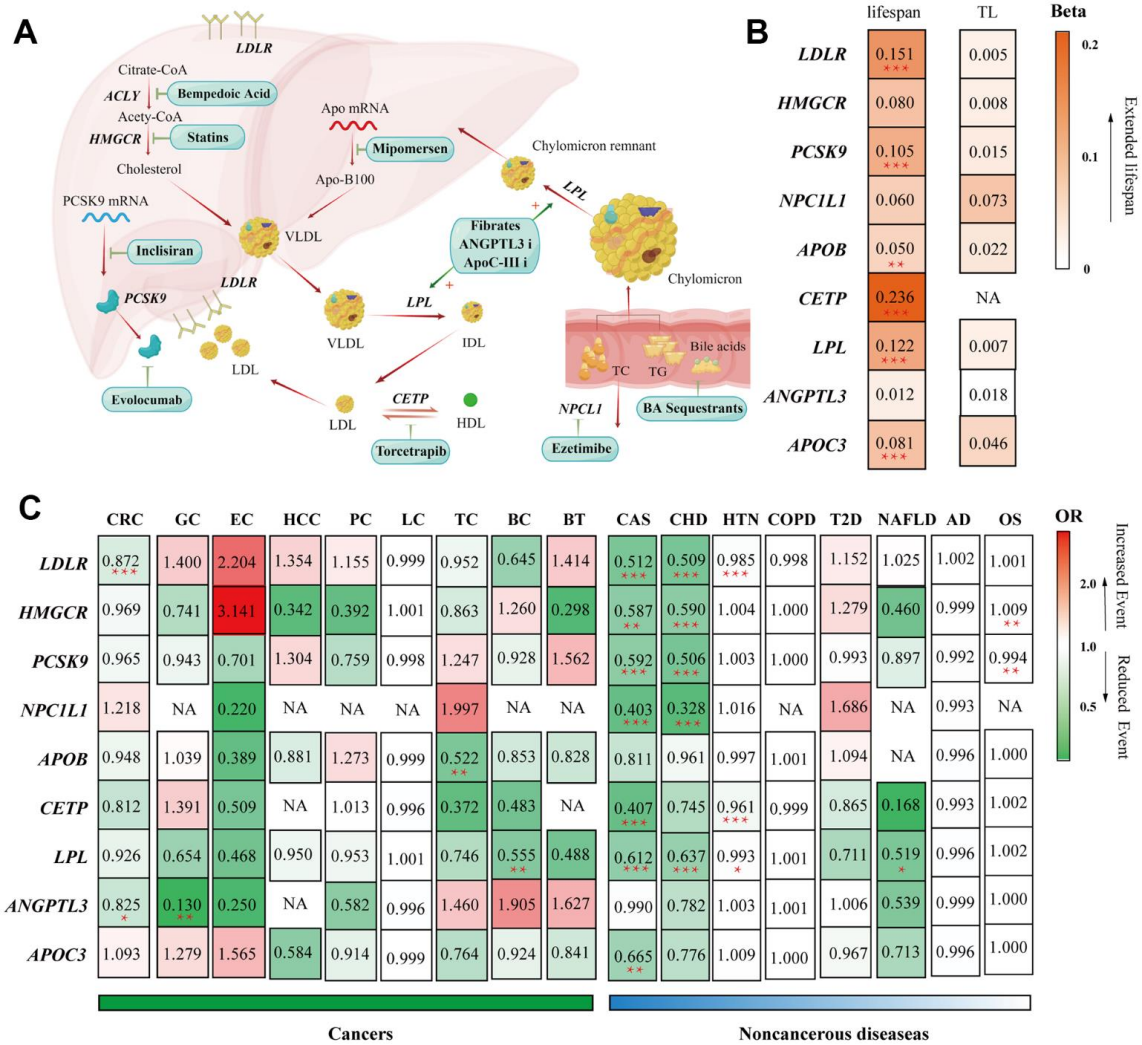
**Summary of the study.** (**A**) Summary of the mechanisms of action of lipid-lowering pharmaceutical agents included in our study. (**B**) Heatmap visualization of the Beta or OR estimates of lipid-lowering drug targets on different outcomes. The figure displays a matrix with rows representing gene targets of lipid-lowering agents and columns representing outcomes from different GWAS consortiums. The values in each square indicate the Beta or OR estimates and are color-coded based on their specific values. (**B**) Heatmap applied the gradually deepening orange, indicating the increasing Beta values. (**C**) Heatmap applied the deepening red indicating the increasing OR values and the deepening green representing the decreasing ORs. Abbreviations: TL, telomere length; CHD, Major coronary heart disease; CAS, Coronary atherosclerosis; T2D, Type 2 diabetes; HTN, hypertension; NAFLD, non-alcoholic fatty liver disease; OS, osteoporosis; CRC, colorectal cancer; GC, gastric cancer; EC, esophageal cancer; HCC, hepatocellular carcinoma; LC, lung cancer; PC, pancreatic cancer; TC, thyroid cancer, BC, bladder cancer; BT, brain tumors; COPD, chronic obstructive pulmonary disease; AD, Alzheimer's disease/dementia, OS, osteoporosis.

All the statistical powers of the MR results are presented in Supplementary Table 9. The main results of the study are summarized in Figures 5, 6. The leave-one-out analyses were presented in Supplementary Figure 1.

## DISCUSSION

Lipid metabolism has been reported to play an important role in the human lifespan and aging process [23]. However, findings were mostly derived from the data of animal models such as shorter-lived yeast, flies, and rodents, as lifespan research involving human subjects requires large amounts of time and cost [24]. By utilizing MR analysis, we were able to directly examine the genetic links between circulating lipids and longevity in humans. In this study, we used drug-targeted MR and identified several newer nonstatin lipid-lowering agents, such as those targeting *LPL*, *ANGPTL3*, and *LDLR*, which were associated with increased human lifespans and decreased risks of several common cancers and cardiometabolic disorders.

*LDLR* and *LPL* are two key modulators in lipid metabolism. In our study, both genes had causal effects on human lifespans. The *LDLR* gene can be affected by statin therapies, because interfering with the hepatic cholesterol synthesis could compensate for the increase in the de novo synthesis of LDLR and cause transport of more LDLR to the hepatocellular membranes [25]. We identified two other genes, *PCSK9* and *CETP*, which were also associated with prolonged lifespans, and these two genes could also affect LDLR expression in lipid metabolism. The *PCSK9* inhibitor prevents LDLR degradation, increases LDLR expression, and ultimately aids in the elimination of circulating LDL-C [26]. The *CETP* inhibitor works by increasing LDLR expression and thus lowering LDL-C levels [27]. As the discovery of new drugs such as PCSK9 or *CETP* inhibitors is still ongoing, their impact on cardiovascular morbidity and mortality, as well as lifespan, remains uncertain. Several clinical trials are currently in progress to determine their efficacy [10, 28]. Therefore, our findings provide promising evidence for developing these novel LDL-C-lowering agents.

In addition to LDL-C-lowering drugs through the LDLR pathway, we found that variants in the genes that encode the targets of TG through the LPL pathway were associated with lower risks of several cardiometabolic diseases. We observed that statin use was associated with increased risks of T2D, which is consistent with previous clinical trials reporting potential T2D risks with long-term statin use [29, 30]. In contrast to *HMGCR* inhibitors, *LPL* targets were associated with reduced risks of cardiovascular events, T2D, hypertension, and NAFLD, indicating the promising roles of developing newer nonstatin therapies through the LPL pathway. To gain a better understanding, it is imperative to focus on large-scale epidemiological and long-term randomized trials to specifically investigate the magnitude of the expected clinical benefit of newer nonstatin therapies.

Another finding in our MR study is the potential association between lipid-lowering genes and colorectal cancer (CRC). Although we did not find a direct causal effect of statin-targeted *HMGCR* on CRC risks, we confirmed that lipid-lowering *LDLR* variants, which are the key downstream genes in cholesterol metabolism, were associated with reduced CRC risks. Previous *in vivo* and *in vitro* studies have demonstrated a potential correlation between lipid metabolism and CRC [31, 32]. These studies have suggested that LDLR could be an important target for CRC chemoprevention. Further investigation is needed to study the role of the *LDLR* gene in preventing CRC. In addition, our findings indicate that targeting the lipid-lowering *ANGPTL3* gene may decrease the risk of CRC. *ANGPTL3* plays a role in lipid metabolism via the LPL pathway. Previous research has shown a correlation between *ANGPTL3* and liver metastasis in CRC [33, 34]. Our results suggest that the TG pathways may also contribute to the development of CRC, and *ANGPTL3* could be considered an additional chemopreventive target for CRC.

As a promising approach, this MR study provided genetic associations regarding statin use and human longevity traits. However, these MR results must be interpreted with caution. First, although statistical powers in most MR results exceed 90%, a few results still lack sufficient statistical powers. Thus, these results still need further validation in larger-scale GWAS data that may be available in the future. Third, our study employed multiple genetic proxies of lipid-lowering drugs, which suggests that the observed causal effects on lifespan may be attributed to genetically proxied drug-target genes rather than the whole impact of a specific drug in the real world. While drug-target MR analysis can provide insight into causal effects, it is important to note that it cannot accurately quantify the clinical benefits. Hence, there is a need for additional high-quality randomized trials or large-scale epidemiological studies.

Our study has several limitations. First, we were unable to investigate the causal effects of bempedoic acid or fibrates on human longevity due to the insufficient numbers of target-specific SNPs in the Global Lipids Genetics Consortium. Second, it is worth mentioning that our findings are limited to individuals of European ancestry and should be verified in other populations. To gain a comprehensive understanding, it is crucial to conduct large-scale epidemiological studies and long-term randomized trials to specifically examine the extent of the anticipated clinical benefits of lipid-lowering drugs on age-related outcomes.

## CONCLUSIONS

Our study provides genetic evidence that newer nonstatin lipid-lowering agents have causal effects on decreased risks of several common cancers and cardiometabolic diseases. These data provide genetic insights into the potential benefits of newer nonstatin therapies. Long-term and large-scale clinical trials should also focus on the efficacy of these newer lipid-lowering drugs on age-related outcomes.

## Supplementary Material

Supplementary Figure 1

Supplementary Table 1

Supplementary Table 2

Supplementary Table 3

Supplementary Table 4

Supplementary Table 5

Supplementary Table 6

Supplementary Table 7

Supplementary Table 8

Supplementary Table 9

## References

[1] Istvan ES, Deisenhofer J. Structural mechanism for statin inhibition of HMG-CoA reductase. Science. 2001; 292:1160–4. 10.1126/science.1059344 11349148

[2] Cholesterol Treatment Trialists’ Collaboration. Efficacy and safety of statin therapy in older people: a meta-analysis of individual participant data from 28 randomised controlled trials. Lancet. 2019; 393:407–15. 10.1016/S0140-6736(18)31942-1 30712900 PMC6429627

[3] Fulcher J, O’Connell R, Voysey M, Emberson J, Blackwell L, Mihaylova B, Simes J, Collins R, Kirby A, Colhoun H, Braunwald E, La Rosa J, Pedersen TR, et al, and Cholesterol Treatment Trialists’ (CTT) Collaboration. Efficacy and safety of LDL-lowering therapy among men and women: meta-analysis of individual data from 174,000 participants in 27 randomised trials. Lancet. 2015; 385:1397–405. 10.1016/S0140-6736(14)61368-4 25579834

[4] Raposeiras-Roubin S, Rosselló X, Oliva B, Fernández-Friera L, Mendiguren JM, Andrés V, Bueno H, Sanz J, Martínez de Vega V, Abu-Assi E, Iñiguez A, Fernández-Ortiz A, Ibáñez B, Fuster V. Triglycerides and Residual Atherosclerotic Risk. J Am Coll Cardiol. 2021; 77:3031–41. 10.1016/j.jacc.2021.04.059 34140107 PMC8215641

[5] Ridker PM, Pradhan A, MacFadyen JG, Libby P, Glynn RJ. Cardiovascular benefits and diabetes risks of statin therapy in primary prevention: an analysis from the JUPITER trial. Lancet. 2012; 380:565–71. 10.1016/S0140-6736(12)61190-8 22883507 PMC3774022

[6] Stroes ES, Thompson PD, Corsini A, Vladutiu GD, Raal FJ, Ray KK, Roden M, Stein E, Tokgözoğlu L, Nordestgaard BG, Bruckert E, De Backer G, Krauss RM, et al, and European Atherosclerosis Society Consensus Panel. Statin-associated muscle symptoms: impact on statin therapy-European Atherosclerosis Society Consensus Panel Statement on Assessment, Aetiology and Management. Eur Heart J. 2015; 36:1012–22. 10.1093/eurheartj/ehv043 25694464 PMC4416140

[7] Raal FJ, Rosenson RS, Reeskamp LF, Hovingh GK, Kastelein JJ, Rubba P, Ali S, Banerjee P, Chan KC, Gipe DA, Khilla N, Pordy R, Weinreich DM, et al, and ELIPSE HoFH Investigators. Evinacumab for Homozygous Familial Hypercholesterolemia. N Engl J Med. 2020; 383:711–20. 10.1056/NEJMoa2004215 32813947

[8] Thompson PD, Rubino J, Janik MJ, MacDougall DE, McBride SJ, Margulies JR, Newton RS. Use of ETC-1002 to treat hypercholesterolemia in patients with statin intolerance. J Clin Lipidol. 2015; 9:295–304. 10.1016/j.jacl.2015.03.003 26073387

[9] Akoumianakis I, Zvintzou E, Kypreos K, Filippatos TD. ANGPTL3 and Apolipoprotein C-III as Novel Lipid-Lowering Targets. Curr Atheroscler Rep. 2021; 23:20. 10.1007/s11883-021-00914-7 33694000

[10] Lloyd-Jones DM, Morris PB, Ballantyne CM, Birtcher KK, Covington AM, DePalma SM, Minissian MB, Orringer CE, Smith SC Jr, Waring AA, Wilkins JT, and Writing Committee. 2022 ACC Expert Consensus Decision Pathway on the Role of Nonstatin Therapies for LDL-Cholesterol Lowering in the Management of Atherosclerotic Cardiovascular Disease Risk: A Report of the American College of Cardiology Solution Set Oversight Committee. J Am Coll Cardiol. 2022; 80:1366–418. 10.1016/j.jacc.2022.07.006 36031461

[11] Davies NM, Holmes MV, Davey Smith G. Reading Mendelian randomisation studies: a guide, glossary, and checklist for clinicians. BMJ. 2018; 362:k601. 10.1136/bmj.k601 30002074 PMC6041728

[12] Evans DS. Target Discovery for Drug Development Using Mendelian Randomization. Methods Mol Biol. 2022; 2547:1–20. 10.1007/978-1-0716-2573-6_1 36068458

[13] Authors/Task Force Members, ESC Committee for Practice Guidelines (CPG), and ESC National Cardiac Societies. 2019 ESC/EAS guidelines for the management of dyslipidaemias: Lipid modification to reduce cardiovascular risk. Atherosclerosis. 2019; 290:140–205. 10.1016/j.atherosclerosis.2019.08.014 31591002

[14] Schmidt AF, Finan C, Gordillo-Marañón M, Asselbergs FW, Freitag DF, Patel RS, Tyl B, Chopade S, Faraway R, Zwierzyna M, Hingorani AD. Genetic drug target validation using Mendelian randomisation. Nat Commun. 2020; 11:3255. 10.1038/s41467-020-16969-0 32591531 PMC7320010

[15] Willer CJ, Schmidt EM, Sengupta S, Peloso GM, Gustafsson S, Kanoni S, Ganna A, Chen J, Buchkovich ML, Mora S, Beckmann JS, Bragg-Gresham JL, Chang HY, et al, and Global Lipids Genetics Consortium. Discovery and refinement of loci associated with lipid levels. Nat Genet. 2013; 45:1274–83. 10.1038/ng.2797 24097068 PMC3838666

[16] Mattioli K, Oliveros W, Gerhardinger C, Andergassen D, Maass PG, Rinn JL, Melé M. Cis and trans effects differentially contribute to the evolution of promoters and enhancers. Genome Biol. 2020; 21:210. 10.1186/s13059-020-02110-3 32819422 PMC7439725

[17] Hemani G, Tilling K, Davey Smith G. Orienting the causal relationship between imprecisely measured traits using GWAS summary data. PLoS Genet. 2017; 13:e1007081. 10.1371/journal.pgen.1007081 29149188 PMC5711033

[18] Hemani G, Zheng J, Elsworth B, Wade KH, Haberland V, Baird D, Laurin C, Burgess S, Bowden J, Langdon R, Tan VY, Yarmolinsky J, Shihab HA, et al. The MR-Base platform supports systematic causal inference across the human phenome. Elife. 2018; 7:e34408. 10.7554/eLife.34408 29846171 PMC5976434

[19] Andrade C. Understanding the Basics of Meta-Analysis and How to Read a Forest Plot: As Simple as It Gets. J Clin Psychiatry. 2020; 81:20f13698. 10.4088/JCP.20f13698 33027562

[20] Staiger D, Stock JH. Instrumental variables regression with weak instruments. Econometrica. 1997; 65:557–86. 10.2307/2171753

[21] Burgess S, Dudbridge F, Thompson SG. Combining information on multiple instrumental variables in Mendelian randomization: comparison of allele score and summarized data methods. Stat Med. 2016; 35:1880–906. 10.1002/sim.6835 26661904 PMC4832315

[22] Timmers PR, Mounier N, Lall K, Fischer K, Ning Z, Feng X, Bretherick AD, Clark DW, Shen X, Esko T, Kutalik Z, Wilson JF, Joshi PK, and eQTLGen Consortium. Genomics of 1 million parent lifespans implicates novel pathways and common diseases and distinguishes survival chances. Elife. 2019; 8:e39856. 10.7554/eLife.39856 30642433 PMC6333444

[23] Mutlu AS, Duffy J, Wang MC. Lipid metabolism and lipid signals in aging and longevity. Dev Cell. 2021; 56:1394–407. 10.1016/j.devcel.2021.03.034 33891896 PMC8173711

[24] Johnson AA, Stolzing A. The role of lipid metabolism in aging, lifespan regulation, and age-related disease. Aging Cell. 2019; 18:e13048. 10.1111/acel.13048 31560163 PMC6826135

[25] Goldstein JL, Brown MS. The LDL receptor. Arterioscler Thromb Vasc Biol. 2009; 29:431–8. 10.1161/ATVBAHA.108.179564 19299327 PMC2740366

[26] Fitzgerald K, Frank-Kamenetsky M, Shulga-Morskaya S, Liebow A, Bettencourt BR, Sutherland JE, Hutabarat RM, Clausen VA, Karsten V, Cehelsky J, Nochur SV, Kotelianski V, Horton J, et al. Effect of an RNA interference drug on the synthesis of proprotein convertase subtilisin/kexin type 9 (PCSK9) and the concentration of serum LDL cholesterol in healthy volunteers: a randomised, single-blind, placebo-controlled, phase 1 trial. Lancet. 2014; 383:60–8. 10.1016/S0140-6736(13)61914-5 24094767 PMC4387547

[27] Dong B, Singh AB, Fung C, Kan K, Liu J. CETP inhibitors downregulate hepatic LDL receptor and PCSK9 expression *in vitro* and *in vivo* through a SREBP2 dependent mechanism. Atherosclerosis. 2014; 235:449–62. 10.1016/j.atherosclerosis.2014.05.931 24950000 PMC4539152

[28] Nicholls SJ, Ditmarsch M, Kastelein JJ, Rigby SP, Kling D, Curcio DL, Alp NJ, Davidson MH. Lipid lowering effects of the CETP inhibitor obicetrapib in combination with high-intensity statins: a randomized phase 2 trial. Nat Med. 2022; 28:1672–8. 10.1038/s41591-022-01936-7 35953719

[29] Sattar N, Preiss D, Murray HM, Welsh P, Buckley BM, de Craen AJ, Seshasai SR, McMurray JJ, Freeman DJ, Jukema JW, Macfarlane PW, Packard CJ, Stott DJ, et al. Statins and risk of incident diabetes: a collaborative meta-analysis of randomised statin trials. Lancet. 2010; 375:735–42. 10.1016/S0140-6736(09)61965-6 20167359

[30] Galicia-Garcia U, Jebari S, Larrea-Sebal A, Uribe KB, Siddiqi H, Ostolaza H, Benito-Vicente A, Martín C. Statin Treatment-Induced Development of Type 2 Diabetes: From Clinical Evidence to Mechanistic Insights. Int J Mol Sci. 2020; 21:4725. 10.3390/ijms21134725 32630698 PMC7369709

[31] Pakiet A, Kobiela J, Stepnowski P, Sledzinski T, Mika A. Changes in lipids composition and metabolism in colorectal cancer: a review. Lipids Health Dis. 2019; 18:29. 10.1186/s12944-019-0977-8 30684960 PMC6347819

[32] Tanaka T, Oyama T, Sugie S, Shimizu M. Different Susceptibilities between Apoe- and Ldlr-Deficient Mice to Inflammation-Associated Colorectal Carcinogenesis. Int J Mol Sci. 2016; 17:1806. 10.3390/ijms17111806 27801847 PMC5133807

[33] Wang Y, Yi Y, Pan S, Zhang Y, Fu J, Wu X, Qin X. Angiopoietin-like protein 3 promotes colorectal cancer progression and liver metastasis partly via the mitogen-activated protein kinase 14 pathway. Mol Carcinog. 2023; 62:546–60. 10.1002/mc.23506 36692110

[34] Cheng J, Song X, Ao L, Chen R, Chi M, Guo Y, Zhang J, Li H, Zhao W, Guo Z, Wang X. Shared liver-like transcriptional characteristics in liver metastases and corresponding primary colorectal tumors. J Cancer. 2018; 9:1500–5. 10.7150/jca.23017 29721060 PMC5929095

