# Supplementary material for: Causal effects of lipid-lowering therapies on aging-related outcomes and risk of cancers: a drug-target Mendelian randomization study
Source: Aging (Albany NY). 2023 Dec 19;15(24):15228–42. doi: 10.18632/aging.205347 (PMC10781452; doi:10.18632/aging.205347)
Supplement: Supplementary Figure 1 [file aging-15-205347-s001.pdf]

SUPPLEMENTARY FIGURE

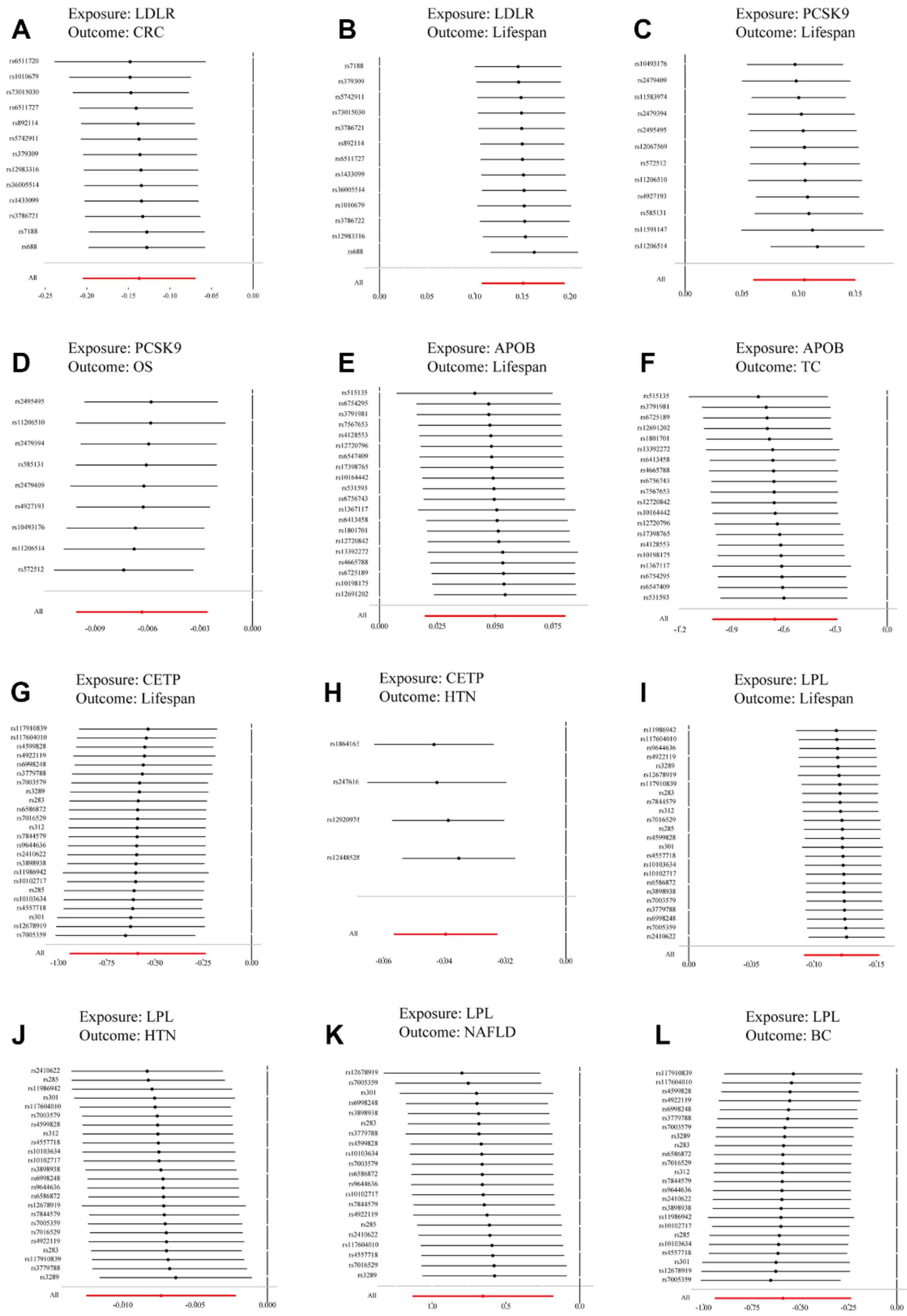

Supplementary Figure 1. Leave-one-out plots for the causal effects of representative lipid-lowering drug target genes (Exposure) that had a causal relationship with the different diseases (outcomes). (**A**) Causal effects of LDLR on CRC; (**B**) Causal effects of LDLR on lifespan; (**C**) Causal effects of PCSK9 on lifespan; (**D**) Causal effects of PCSK9 on OS; (**E**) Causal effects of APOB on lifespan; (**F**) Causal effects of APOB on TC; (**G**) Causal effects of CETP on lifespan; (**H**) Causal effects of CETP on HTN; (**I**) Causal effects of LPL on lifespan; (**J**) Causal effects of LPL on HTN; (**K**) Causal effects of LPL on NAFLD; (**L**) Causal effects of LPL on BC.
