# Supplementary material for: Causal effects of lipid-lowering therapies on aging-related outcomes and risk of cancers: a drug-target Mendelian randomization study
Source: Aging (Albany NY). 2023 Dec 19;15(24):15228–42. doi: 10.18632/aging.205347 (PMC10781452; doi:10.18632/aging.205347)
Supplement: Supplementary Table 9 [file aging-15-205347-s010.pdf]

Supplementary Table 9. Statistical power for drug-target MR analyses.

Drug target	R2	Statistical power											
		Lifespan	CAS	CHD	T2D	Hypertension	NAFLD	Osteoporosis	CRC	GC	EC	TC	BC
LDLR	0.11%	98%	92%	100%	100%	99%	12%	90%	100%	91%	100%	61%	33%
HMGCR	0.10%	74%	95%	100%	100%	96%	6%	99%	100%	38%	100%	48%	80%
PCSK9	0.15%	72%	98%	100%	100%	100%	10%	96%	100%	67%	43%	89%	66%
NPC1L1	0.06%	16%	68%	100%	100%	93%	-	-	100%	-	64%	-	-
APOB	0.14%	86%	100%	100%	100%	99%	25%	100%	100%	93%	25%	41%	80%
CETP	0.08%	18%	63%	99%	100%	91%	6%	100%	99%	87%	20%	13%	19%
LPL	0.16%	96%	82%	93%	100%	98%	19%	100%	100%	31%	13%	26%	16%
ANGPTL3	0.19%	10%	100%	98%	100%	88%	6%	100%	100%	24%	68%	100%	100%
APOC3	0.19%	70%	100%	99%	100%	99%	20%	100%	100%	21%	13%	26%	16%
